# Experimental approaches to assess melanocytes mosaicism in segmental vitiligo^☆^

**DOI:** 10.1016/j.abd.2022.05.001

**Published:** 2022-12-16

**Authors:** Gerson Dellatorre, Vinicius M. Fava, Marcelo Távora Mira, Caio Cesar Silva de Castro

**Affiliations:** a Santa Casa de Misericórdia Hospital, Curitiba, PR, Brazil; b Infectious Diseases and Immunity in Global Health Program, The Research Institute of the McGill University Health Centre, Montreal, QC, Canada; c Graduate Program in Health Sciences, School of Medicine, Pontifícia Universidade Católica do Paraná, Curitiba, PR, Brazil; d School of Life Sciences, Pontifícia Universidade Católica do Paraná, Curitiba, PR, Brazil; e School of Medicine, Pontifícia Universidade Católica do Paraná, Curitiba, PR, Brazil

**Keywords:** Genetics, Mosaicism, Vitiligo

## Abstract

Vitiligo is an autoimmune disease of the skin that results in localized or disseminated white macules. One common feature of several existing classification protocols is the distribution of the disease into two main subtypes, non-segmental vitiligo (NSV) and segmental vitiligo (SV). SV is characterized by depigmentation spreading within one or more skin segments while NSV is widespread. Several clinical-epidemiological observations suggest that SV has distinct autoimmune pathophysiology compared to NSV. Furthermore, the clinical distribution pattern of SV lesions closely resembles other melanocyte mosaicism diseases. These observations led us to hypothesize that SV is caused by a localized autoimmune reaction targeting epidermal mosaicism melanocytes. Here, we proposed examples of experimental approaches to assess mosaicism in SV patients.

## Introduction

According to an international consensus, vitiligo is classified into three main groups: Segmental (SV), Non-Segmental (NSV), and mixed vitiligo (coexistence of SV and NSV).^1^ The consensus describes SV as a depigmentation spreading within a segment, uni-, bi-, or pluri-segmental, however, this distribution can occasionally be bilateral.^1^, ^2^ The segmental distribution pattern is not the only difference between SV and NSV (Table 1). The median age of SV onset is 16 years, on average 8 to 10 years earlier than the median age of NSV onset.^3^, ^4^, ^5^ Additionally, depigmentation in SV typically has a rapid progression with a limited-time course between 6 to 24 months, rarely extending after this period, while NSV is chronic with an uncertain lifelong time course.^1^, ^6^ In contrast to NSV, SV presents early involvement of hair follicles melanocytes, with up to 50% of SV patients exhibiting poliosis in the affected area.^1^ The prevalence of concomitant autoimmune disorders (e.g., thyroiditis) is lower in SV.^3^, ^7^ SV and NSV also differ regarding response to treatment: in general, SV patients have a poor response to phototherapy compared to NSV, possibly due to earlier depletion of follicular reservoir in the former.^8^, ^9^ Conversely, SV patients have an excellent and long-term response to surgical interventions such as melanocyte-keratinocyte transplant.^10^, ^11^ The long-term success of transplant therapy in SV suggests a confined defect of melanocyte–keratinocyte metabolism.^10^

**Table 1:** Characteristics of segmental and non-segmental vitiligo.

	Segmental	Non-segmental
Median age of onset	Earlier onset	Later onset
Association with autoimmune diseases	Less frequent	Frequent
Role of early involvement of oxidative Stress	Unknown	Present
Clinical Manifestation	Segmental, unilateral	Varied
Course	Short and limited	Chronic and unstable
Serum TWEAK levels	Higher	Lower
Tregs	Unaffected	Diminished
Melanocyte depletion at the follicle (polyosis)	Frequent	Less frequent
Response to clinical therapies	Poor	Mod / Good
Long-term response to melanocyte-keratinocyte transplant procedure	Better	Worse

Reports suggest differences in the biological mechanisms underlying the pathogenesis of SV as compared to NSV. For example, serum levels of TWEAK (Tumor Necrosis Factor-like Weak inducer of Apoptosis) were significantly higher in SV compared to NSV patients.^12^ Moreover, TWEAK was shown as a biomarker with 100% sensitivity and 80.1% specificity in differentiating SV from NSV.^12^ In contrast to NSV, systemic oxidative stress has a weak and limited contribution to SV pathogenesis.^13^, ^14^ In SV, a significant increase of stress-induced markers (e.g., mitochondrial, HSP70 and CXCL16) was observed only in perilesional skin suggesting a localized pathogenic mechanism promoting depigmentation. Immunophenotypic analysis of circulating immune cells of SV patients identified unaltered regulatory T-cells (Tregs) compared to healthy controls while NSV patients had decreased levels of Tregs.^15^ Collectively, multiple pieces of evidence indicate unaltered systemic immunity in SV patients and point to a localized cytotoxic reaction targeting epidermal melanocytes.^15^ Taken together, these differences and the remarkable similarity of SV distribution pattern to mosaic melanocyte diseases (as segmental lentiginose and verrucous epidermal nevus) led us to hypothesize the involvement of somatic mosaicism in SV pathogenesis.^16^, ^17^

## How to test the hypothesis

Mosaicism designates individuals encompassing at least two cell populations derived from a single zygote but with distinct genotype or epigenetic profiles.^18^ The phenotypic presentation of a disease caused by genetic mosaicism is conditional to the type of variation and phase of development that a somatic mutation that occurred.^19^ Variants leading to genetic mosaicism range from chromosomal duplications, segment translocations, copy number variation (CNV), single-nucleotide variants (SNV), or epigenetic changes such as transcriptomic alterations caused by retrotransposition insertions. The embryonic phase and cell differentiation status where a *de novo* mutation or retrotransposition occurred delineates the extent of tissues/cells involved in the mosaicism. The hypothesis to be tested suggest that genetic mosaicism in SV occurred at some point during skin/melanocyte differentiation. Different approaches can be applied to test the mosaicism hypothesis in SV, each aiming to detect a distinct type of mosaicism. Here we proposed examples by adapting designs applied to study the host response to infections, detect somatic mutations in cancers and evaluate embryonic development.^20^, ^21^, ^22^, ^23^

### Paired contrast of perilesional skin and contralateral healthy skin in SV

**Figure 1:**
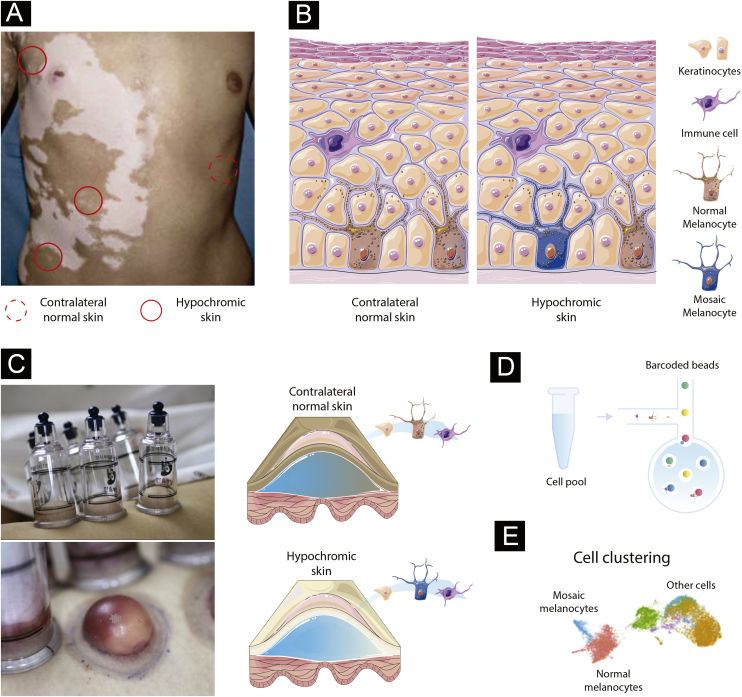
Experimental design to test the mosaicism hypothesis in segmental vitiligo. (A) Depiction of a segmental vitiligo patient. Hypochromic areas highlighted with a full red circle would be evaluated for the presence of mosaic melanocytes. A contralateral skin sample marked with a dotted circle would be used as an internal control. (B) Skin composition for contralateral and hypochromic skin. The hypochromic skin includes residual mosaic melanocytes. (C) Subepidermal suction blister technique of normal and affected skin. The trypsinization of the blister’s roof allows the detachment of keratinocytes and melanocytes that can be together with immune infiltrated cells used in the single-cell experiments. (D) Single-cell barcoding. The pool containing cell suspension for normal and affected skin would be loaded to the single-cell channel. Individual cells would be incorporated into oil droplets and marked with unique barcoded beads, which would allow the application of different sets of single-cell approaches including scWGS, scRNA, and scATAC. (E) Cell clustering using omics data. Clustering analysis would group cells sharing similar states and identify mosaic melanocytes as well as other cell types included in the blister extract.

The detection of mosaicism in human diseases can be challenging as the number of mosaic cells within the targeted tissue might be small. In addition, mosaic melanocytes would likely be absent inside existing SV lesions as a loss of melanocytes is the cause of vitiligo. Therefore, high-resolution methods at the single-cell level may be required to detect underrepresented cell populations.^24^ To detect potential mosaic cells, evaluating tissue obtained from hypochromic skin regions from individuals at the earliest stage of SV would be required (Fig. 1A). An advantage of studying the mosaicism hypothesis in SV is the possibility to use internal controls since SV is usually constrained to a unilateral segment: if the *de novo* mutation occurred during skin differentiation in one segment, a contralateral healthy skin could be used to establish the melanocyte’s “normal” profile (Fig. 1A). This strategy would allow controlling for confounding effects caused by interindividual variability when combining multiple SV cases.^25^

### Target tissue preparation

Melanocytes account for ∼2.8% of the cell population in the epidermis and approximately 1200 melanocytes exist per mm^2^ of the skin independently of an individual ethnicity (Fig. 1B).^26^ To capture a representative number of viable mosaic melanocytes for the single-cell analysis, hypochromic areas of early-onset active SV would need to be determined using Wood’s lamp. Keratinocyte-melanocyte sampling of these areas could be performed with a suction blister epidermal grafting technique with subsequent trypsinization of the blister’s roof to detach the cells into a suspension (Fig. 1C).^27^ An advantage of collecting epidermis by using the suction blister is the widening of the sampled area, which increases the likelihood of capturing remaining viable mosaic melanocytes while being nearly a scarless technique, which facilitates patient inclusion.^28^ To evaluate follicular melanoblasts not captured by the blister method, a punch technique could also be used. A punch biopsy is more invasive than blistering and the overall proportion of melanoblasts captured would be small. However, methodological refinements of single cell analysis could allow in a near future the study of smaller cell populations.

### Identifying mosaic cells in segmental vitiligo via genetic and epigenetic variations

To evaluate the presence of mosaic melanocytes, fluorescence-activated cell sorting selection could be used to separate the melanocyte fraction of blister extracts or melanoblasts from punch biopsies. Next, whole genome sequencing (WGS) would be performed using DNA extracted from melanocytes from hypochromic and contralateral skin from the same individuals. Variants detected in contralateral tissues would be used to exclude germline variants. Algorithms designed to detect tumor cells, such as MuTect2,^29^ or specific for mosaic cells, such as MosaicForecast^30^ and DeepMosaic,^31^ could be used to detect somatic mutations. The limitation of the bulk approach is the inability to separate mosaic cells from regular melanocytes and evaluate their interaction with other cells. Addressing this limitation would require single-cell (sc) sequencing technologies evaluating either the transcriptomic, epigenetic, or DNA sequence profile of individual cells (Fig. 1D).^32^ scWGS allows the evaluation of structural variation, CNVs, and SNVs in individual cells. A high throughput scWGS method developed to detect subclonal mutations in cancer could be used to test the hypothesis of melanocyte mosaicism.^22^ In SV, mosaic cells would share a state (i.e., specific genetic variants) that could be used to merge cells in clusters and define the proportion and the type of genetic variants present in mosaic cells (Fig. 1E).^33^ Epigenetic mosaicism in SV could be tested by using a multi-omics approach with transcriptomic (scRNA) and chromatin accessibility (scATAC) measures. Multi-omics approaches are currently being used to study a diverse set of diseases.^34^ Analyses of scRNA and scATAC could be used to identify mosaic cells via transcriptomic and epigenomic similarities with tools such as Seurat,^35^ Monocle,^36^ and Cicero.^37^ A similar scRNA approach applied to study human embryos was able to successfully detect mosaicism.^20^ An advantage of the single cell compared to bulk approaches is the ability to study the relationship between cells present in the blister extract or punch biopsies. In fact, scRNA study of blistering extracts has shown the recovery of melanocytes from NSV lesions.^24^ Applying a single-cell approach to study SV could test the mosaicism hypothesis while assessing the local immune profile as shown for NSV.^24^

## Financial support

None declared.

## Authors’ contributions

Gerson Dellatorre: Study concept, writing and approval of the final manuscript.

Vinicius Medeiros Fava: Study concept, writing and approval of the final manuscript.

Marcelo Távora Mira: Writing and approval of the final manuscript.

Caio Cesar Silva de Castro: Study concept, writing and approval of the final manuscript.

## Conflicts of interest

None declared.
